# Suspension Trauma: A Clinical Review

**DOI:** 10.7759/cureus.8514

**Published:** 2020-06-08

**Authors:** Sean A Weber, Mackenzie M McGahan, Christoph Kaufmann, Saptarshi Biswas

**Affiliations:** 1 Surgery, Grand Strand Medical Center, Myrtle Beach, USA; 2 Trauma and Acute Care Surgery, Allegheny Health Network, Pittsburgh, USA

**Keywords:** suspension trauma, suspension syndrome, harness hang syndrome, harness, reflow syndrome, rescue death, orthostatic syndrome, orthostatic intolerance, fall protection

## Abstract

Suspension trauma is a potentially dangerous outcome of the body’s normal physiological response to motionless vertical suspension from a rope. All who use a safety harness are at risk, and the growing need for occupational work at extreme heights in addition to the interest in caving and mountaineering worldwide has led to an increased number of individuals wearing protective harnesses for work and recreation. It has been described as the cause of death in many climbing incidents and has been demonstrated in multiple studies for improvement of employee fall protection.

Although suspension trauma is potentially life-threatening, there is a lack of scientific data to define an exact mechanism of injury, and there is little conclusive evidence about the proper management of victims. This has led to controversy surrounding the topic of postsuspension management. The discussion of suspension trauma has historically been led by nonmedical experts, but the recent push for more evidence-based research has created a better understanding of the condition. Further investigation of the pathophysiological mechanism of suspension trauma and more complete collection of data from individual cases will increase our understanding of the topic and lead to better decision making in the management of victims.

## Introduction and background

Suspension trauma, also known as harness hang syndrome, is the unfavorable outcome of the human body’s normal physiological response to hanging in a stagnant vertical position for a prolonged period of time [1]. For the last 50 years, this topic has been of particular interest to certain members of the emergency medicine community due to controversy surrounding the efficacy of various rescue techniques and the proper management of patients who have been suspended from a harness for any significant time period [1]. Current points of debate include the most effective type of harness, body positioning following rescue, and the possibility of reperfusion injury after victims are rescued.

Although it is a relatively unknown condition in the medical field as a whole, avid adventurists and many construction employees are at risk of life-threatening injury due to suspension trauma [2]. In fact, any occupation or hobby that requires the use of a harness puts the user at risk. This especially includes the novice rock climber utilizing an ill-fitted harness for recreation and individuals at work who have not been adequately trained regarding suspension equipment and safety. In addition to modern-day interest in the matter, suspension trauma has also been proposed as a probable cause of death in individuals who were crucified during ancient Roman times. Unfortunately, this theory is unlikely to be confirmed due to limited archeological evidence [3].

There is a relative paucity of published information on suspension trauma, and until 2006 many database searches returned zero results on the topic [4]. An extensive database search for suspension trauma in 2009 yielded only five medical articles associated with suspension trauma and harness suspension. However, a complete search of the internet identified nearly 21,000 hits with articles written worldwide by adventurists and experts in fall protection, commercial industry, occupational safety, climbing, and caving [5]. Due to the shortage of scientific data and a large number of opinions, management of suspension victims after their rescue has historically been a controversial topic. 

The purpose of this review is to explain the pathophysiology, elucidate the most current treatment recommendations, and increase awareness of suspension trauma as a whole.

## Review

Etiology

Inherent to any occupational or recreational activity performed at significant height is the risk of serious injury due to falling. For this reason, many safety rules and regulations have been created in order to protect against this risk, including the use of a personal fall arrest system. One component of these systems is the harness, typically worn around the shoulders and upper thighs in order to distribute force away from vital organs in the event of a fall. This is in contrast to the previously used safety belt system which would essentially direct the entire weight of the fall towards the abdomen or chest, resulting in poor outcomes such as injuries to the spine or respiratory compromise [6-7]. Another purpose of new harness designs is to keep the individual upright, maintaining the spine in a vertical position to best absorb the forces experienced during a fall from height [8]. Although the harness is effective in preventing immediate injury, remaining airborne in the harness for a duration of time can result in suspension syndrome, and the rescue mechanism itself can become the source of morbidity and mortality.

Pathogenesis

The mechanism behind suspension trauma is complex and is not as simply understood as its often used synonym, ‘orthostatic intolerance,’ may infer. There are many variables that come into play which determine both the severity of injury as well as the potential to recover following an episode of suspension from a harness [9]. These will be discussed here.

The first aspect of suspension syndrome that must be understood is the significance of the victim being completely motionless while suspended in the air. In a healthy, awake, and alert individual, contraction of musculature in the lower limb compresses the veins, serving as a constant pump to make use of the one-way valves in these vessels [10]. Muscle contraction is necessary in order to return deoxygenated blood against gravity to the vena cava and eventually to the right side of the heart. This muscular pump is so effective that while lower limb muscles are active during walking, venous pressure in the foot is normally around 25 mmHg, but while standing perfectly still the venous pressure in the foot can exceed 90 mmHg [11]. When the pump is disrupted due to the victim being immobilized, blood is not able to return to the heart and will pool in the lower limbs. This results in a decrease in cerebral perfusion and a likely syncopal episode. Studies report that greater than 20% of circulating blood volume can be confined to the lower extremities, resulting in an equal decrease in cardiac output due to a relative hypovolemia [12]. Therefore, a victim who is left hanging in their harness and unable to provide muscle contractions is much more susceptible to the effects of venous pooling than one who is able to move spontaneously. 

A randomized crossover trial performed by Simon et al. in 2019 was the first of its kind as the investigators assessed venous pooling in participants suspended from a harness. The study demonstrated a marked decrease in oxygen saturation (O2Sat) in the lower leg with concomitant cyanosis of the feet in almost all participants. Also, it was found that the diameter of the superficial femoral vein (SFV) increased immediately after the suspension was initiated but failed to progress in size as time went on, likely because the veins were already maximally distended. The gradual decrease in O2Sat combined with the unchanging diameter of the SFV is an evidence of blood pooling in the small veins of the leg. Ultrasound demonstrated no limitation of flow in the arterial vessels of the leg, further proving that the decline in lower limb O2Sat was due to the accumulation of desaturated venous blood rather than lack of oxygen supply [2]. The condition may be worsened by the fact that arterial blood is still able to be pumped into the lower extremities, but is unable to return. 

Along with immobility, the sustained vertical position assumed by victims of suspension syndrome must also be considered when discussing the mechanism of bodily harm. An individual who experiences lower extremity venous pooling during prolonged standing may experience a syncopal episode, lose consciousness and collapse, becoming horizontal on the ground. As a result, a minimal amount of time is spent in the vertical position while the person is unconscious. This is important to recognize because barring any injuries from the fall, recovery will likely be rapid and uneventful as venous blood from the lower extremities, no longer fighting against gravity, can return to the heart and reperfuse the brain [11]. In comparison, a person suspended from a harness above the ground will remain in a vertical position following a syncopal episode. They will not experience the compensatory move into a horizontal position, and venous blood will remain pooled in the lower extremities as described above. 

In addition to understanding the impact of vertical positioning and immobilization, it is important to consider the many possible events that can lead a person to become suspended motionless while wearing a harness in the first place. In most instances suspension syndrome is preceded by an episode of pain, hypoglycemia, injury, fatigue, hypothermia, or fear, causing the individual to fall [2, 4]. After the initial fall, the victim becomes suspended from their harness. Trapped in a vertical position, blood begins to pool in the lower legs, cardiac output decreases, cerebral perfusion declines, and the victim begins to have presyncopal symptoms such as dizziness, lightheadedness, diaphoresis, numbness in the extremities, changes in vision, or nausea. Many study participants have even experienced loss of consciousness during a suspension lasting as little as seven minutes [13]. These symptoms are likely due to the decreased cardiac output associated with relative hypovolemia from lower extremity venous blood pooling. Related research has shown that similar symptomology can be reproduced with table tilt testing in up to 87% of healthy participants using a 50-degree head tilt, and that pain is an independent trigger for syncopal episodes [2, 14].

The specific type of fall arrest system being used is another important consideration as there are unique contributing factors to the pathophysiology of suspension syndrome depending on the type of harness utilized. Prior to the first studies on the topic in 1972, most systems included either a belt around the chest or a belt around the waist. The initial waist belts caused considerable trauma during a fall because all of the forces of the falling person were directed towards their abdomen. Without any redistribution of weight, fall victims could suffer serious injury to internal organs or spine [6-7]. It was later discovered that a harness around the chest alone during suspension could result in increased intrathoracic pressure, leading quickly to compromise of pulmonary function [10]. The most commonly used harnesses now include straps around the shoulders as well as straps around the upper thighs which help to keep the spine in a vertical position to best absorb the impact of the falling victim’s weight. This has proven to be effective in preventing injury from the initial fall; however, it is likely that during prolonged suspension the femoral veins are compressed by the harness straps in the groin. This can further exacerbate the pooling of venous blood in the lower limbs [4]. As hydrostatic pressure increases within the vessels, fluid is forced into the interstitium, and further edema can accumulate in the legs. In response to the apparent decrease in blood volume, arterial and venous tone is reflexively increased to increase systemic vascular resistance and maintain adequate blood pressure. This compensatory mechanism results in even greater pressure against the harness straps, further contributing to lower extremity edema and decreased cerebral perfusion [11].

Management recommendations

Although the immediate management of suspended victims has historically been a controversial topic, recent evidence and professional opinions are beginning to converge. Rescue of the victim should take place as soon as possible safely and the individual brought to a supine position on the ground. Claims to the contrary have been popularized by laypersons, but there is currently no scientific evidence that return to a horizontal position can increase the risk of rescue death [6]. Initial treatment should consist of airway, breathing, circulation (ABC) and a full physical examination including skeletal survey and neurological assessment like one would complete for any trauma patient. If rapid descent is possible the victim should be released immediately irrespective of the clinical status (safe/unresponsive/injured) to avoid the onset of suspension trauma. If rapid descent is not possible and the victim is conscious and not injured, then first responders should stimulate muscular activity in the lower extremities while awaiting backup. This can be achieved by moving the legs, using rescue pedals or lifting the feet against a wall. Advanced Trauma Life Support (ATLS) should be followed and the victim hospitalized regardless of symptom severity [2, 4-5, 10-11]

Reflow syndrome and rescue death: the controversies 

The most controversial issue associated with suspension syndrome is the question of how to proceed after the victim is safely rescued from prolonged suspension and returned to the ground. The phenomenon known as “rescue death” was introduced at the 1972 Innsbruck Conference on Mountain Medicine and has been a hotly debated topic for the last several decades [13]. The theory was that there may be an increased risk of death if victims were returned to a supine position too quickly following prolonged suspension from a harness [3]. This idea was not founded on medical evidence; rather it was based on expert opinion and case reports of climber deaths that occurred following rescue from a period of free suspension [5]. Eight climbers who had not suffered injuries during their initial fall experienced hanging from various harness types for time periods ranging from thirty minutes to eight hours. Following their rescue and return to the ground, all eight of these climbers died over the next 11 days regardless of the time spent in suspension, harness type, and interventions [11]. The fact that none of the climbers had any evidence of traumatic injury and all were returned to the ground safely without complication pointed to either suspension or the rescue method as the cause of death.

The theoretical concept of rescue death was developed based on a number of potential concerns related to the rapid change in position of the victim from vertical to supine. It was recognized that the return of blood to the brain was essential but that there were conceivable dangers that may occur while doing so [11]. First, the large quantity of deoxygenated blood that had accumulated in the legs during a time of vertical immobility could return suddenly to the heart carrying toxic breakdown metabolites and cause cardiac ischemia. Furthermore, it was hypothesized that the rapid return of blood could result in volume overload of the right ventricle, resulting in cardiac arrest. In addition to compromised heart function, reperfusion injury to other vital organs was also considered [4, 10]. End organ damage secondary to rhabdomyolysis is another proposed cause of injury following prompt return to a horizontal position, with the most common renal being kidney failure [7, 11]. 

In 2002, a major review into harness suspension conducted by the Health and Safety Executive of the United Kingdom reiterated the concerns highlighted at the 1972 Innsbruck Conference. This document strongly discouraged horizontal positioning after rescue from a vertical suspended position of any length stating initially “the accident victim must never be laid down” [11]. Instead it suggested placing victims in a seated position with chest above the legs, knees bent, and gradually transferring them to a horizontal position in order to minimize the risk of rescue death. After achieving the seated position, continuous monitoring of respiration and circulation was suggested in addition to inspecting the victim for any signs of trauma or neurological deficits. Hospitalization was also recommended no matter how mild the patient’s condition [11].

During the next decade much work was done to further investigate proper management of victims of suspension trauma. In June 2011 a clinical update was published by Pasquier et al. which found no evidence that returning victims to a horizontal position increased the risk of death [4, 10]. In the same year, Mortimer’s review of suspension trauma also refuted the idea of anoxic blood as a cause of rescue death. He explained that transient acidemia is often created by tourniquets during surgery, but once the tourniquet is released, acidic blood returning to the heart causes only mild cardiac dysfunction. Although the blood in a victim of suspension syndrome may become more acidotic than a surgical patient’s, it is felt to be unlikely that the return of this blood to the heart will cause cardiac death [7]. 

Until 2019, no medical articles offered any conclusive evidence as to whether or not the horizontal position contributes to rescue death and there was no consensus as to the best initial management for victims of suspension trauma. An experimental randomized crossover trial performed by Rauch et al. discovered that cardiac ventricular overdistention did not occur when participants were brought immediately to supine position following prolonged suspension. In addition, all participants in that trial who experienced pre-syncopal events while suspended showed rapid recovery when placed into the supine position [2]. 

The most recent scientific findings are meaningful, but studies on suspension trauma are limited in human subjects. In order to avoid harm, each study to date has used pre-syncopal symptoms as a stopping point for suspension, so it is difficult to determine what might happen to victims who are not able to be rescued immediately.

Prevention

It should be noted that although potentially fatal, the incidence of suspension trauma is very low. Over an 11-year span there were 5.8 million hours recorded “on the rope” by qualified technicians using a harness in various lines of work. Throughout this period there were no reports of syncopal episodes and no incidents of injury due to prolonged suspension [4]. The only reported episodes of suspension trauma during the same time frame were from healthy participants in studies and sporting accidents [10-11]. This is likely due to strong efforts from employers to ensure the safety of their employees.

The first step in prevention of suspension syndrome is to ensure proper equipment that is also well-fitting for each individual as a correctly adjusted harness can likely delay the onset of adverse symptoms [15]. Chest harnesses should be completely avoided due to increased risk of respiratory depression [6]. Instead, straps around the shoulders, waist, and sub-pelvic leg straps should be present as well as adjustable foot loops. Also known as relief straps, foot loops provide added support under the feet and can be adjusted to keep the legs slightly bent, minimizing venous pooling in the legs. These straps also alleviate pressure on the upper thighs and can decrease pain if a victim experiences prolonged suspension [4, 11, 16].

Before using the harness in a live situation, suspension testing should be carried out in a controlled environment so that adjustments to fit can be made if needed. During this time the individual should also be made aware of the potential dangers of prolonged immobile suspension and the mechanism of injury. Recent studies demonstrate an unpredictable time between suspension and onset of pre-syncopal symptoms, so thorough education must be provided regarding the warning signs that precede suspension trauma [2]. It should also be emphasized that the timing of symptom development is important, and these signs indicate the possible rapid development of a life-threatening situation [2, 11]. 

Along with individual training, larger systems should be in place for rescue and initial management of suspended persons in agreement with current evidence. These plans must be detailed and well-rehearsed by emergency personnel. The Occupational Safety and Health Administration (OSHA) currently promotes employee awareness and recognition of risks associated with prolonged suspension [8]. OSHA mandates that those with an occupation necessitating use of a harness be trained in using fall arrest systems and related personal protective equipment [1, 8-9]. Individuals using a harness at height for any reason should never be left alone. This ensures prompt recognition of a fall or syncopal event and can lead to early activation of a rescue plan [4].

In the event that an individual finds him or herself suspended from a harness, they must be trained to pump their legs and to provide lower limb support until rescue is available. If possible, the victim should attempt to press their legs against a firm structure and utilize their foot straps. If these are not accessible, an attempt should be made to keep the legs moving or place them into a more horizontal position to slow the development of pre-syncopal symptoms [1, 4]. During an experiment of suspension with the legs elevated to a horizontal position, only one participant experienced pre-syncopal symptoms, further emphasizing the importance of positioning [14]. By engaging the muscular pump of the legs and providing support, the person can better maintain adequate circulation and delay the onset of suspension trauma [3-4, 13]. All attempts should then be made by rescue personnel to release the victim from the suspended position as soon as it is safely possible. 

Avenues for future research

To date, studies investigating the mechanisms of suspension trauma have used only one type of harness. There is a good reason for future studies to include multiple harness models so that an evidence-based comparison can be made between them. In addition, there should be further work dedicated to creating a more comfortable harness. As pain is a common trigger for syncopal episodes, it would be useful to test different configurations and materials to create a harness that minimizes pain during suspension.

Many of the deaths following suspension trauma have been due to suspension for several hours. There is a good opportunity for investigation as to whether or not interval to rescue should impact management after rescue. It is possible to position victims of prolonged suspension differently after rescue than those who were suspended for a shorter time.

Another consideration is that many climbing accidents resulting in immobile suspension occur at high altitude, creating a situation where hypothermia and further hypoxia may exacerbate the condition. It may be valuable to compare studies that take place at sea level vs. higher elevations.

## Conclusions

Suspension trauma is not well understood in the medical community due to lack of current data. This article reviewed the existing literature to aggregate current evidence regarding pathogenesis, initial management, and prevention. The low incidence of suspension trauma is likely due to proper training in fall arrest systems, good equipment, and well-organized plans for rescue. The emphasis should be on prevention rather than treatment, as it is difficult to study this injury mechanism in humans. Modern harnesses with utilization of foot loops may significantly reduce the incidence of suspension trauma. Much of the controversy surrounding the topic stems from a general lack of scientific evidence and has been driven by a small number of case reports and limited prospective human experiments. However, it can be agreed upon that prompt rescue is key in prevention of morbidity and mortality. The protocol for management of victims after prolonged suspension should be standardized and based on the most recent data. Current evidence suggests immediately placing the victim in the supine position following rescue.
